# Associations between blood pressure across adulthood and late-life brain structure and pathology in the neuroscience substudy of the 1946 British birth cohort (Insight 46): an epidemiological study

**DOI:** 10.1016/S1474-4422(19)30228-5

**Published:** 2019-10

**Authors:** Christopher A Lane, Josephine Barnes, Jennifer M Nicholas, Carole H Sudre, David M Cash, Thomas D Parker, Ian B Malone, Kirsty Lu, Sarah-Naomi James, Ashvini Keshavan, Heidi Murray-Smith, Andrew Wong, Sarah M Buchanan, Sarah E Keuss, Elizabeth Gordon, William Coath, Anna Barnes, John Dickson, Marc Modat, David Thomas, Sebastian J Crutch, Rebecca Hardy, Marcus Richards, Nick C Fox, Jonathan M Schott

**Affiliations:** a Dementia Research Centre, University College London Queen Square Institute of Neurology, University College London, London, UK; b Department of Medical Statistics, London School of Hygiene and Tropical Medicine, University of London, London, UK; c School of Biomedical Engineering and Imaging Sciences, King's College London, London, UK; d Medical Research Council Unit for Lifelong Health and Ageing at University College London, London, UK; e Institute of Nuclear Medicine, University College London Hospitals, London, UK; f Leonard Wolfson Experimental Neurology Centre and Academic Neuroradiological Unit, Department of Brain Repair and Rehabilitation, University College London Queen Square Institute of Neurology, University College London, London, UK; g UK Dementia Research Institute at University College London, University College London, London, UK

## Abstract

**Background:**

Midlife hypertension confers increased risk for cognitive impairment in late life. The sensitive period for risk exposure and extent that risk is mediated through amyloid or vascular-related mechanisms are poorly understood. We aimed to identify if, and when, blood pressure or change in blood pressure during adulthood were associated with late-life brain structure, pathology, and cognition.

**Methods:**

Participants were from Insight 46, a neuroscience substudy of the ongoing longitudinal Medical Research Council National Survey of Health and Development, a birth cohort that initially comprised 5362 individuals born throughout mainland Britain in one week in 1946. Participants aged 69–71 years received T1 and FLAIR volumetric MRI, florbetapir amyloid-PET imaging, and cognitive assessment at University College London (London, UK); all participants were dementia-free. Blood pressure measurements had been collected at ages 36, 43, 53, 60–64, and 69 years. We also calculated blood pressure change variables between ages. Primary outcome measures were white matter hyperintensity volume (WMHV) quantified from multimodal MRI using an automated method, amyloid-β positivity or negativity using a standardised uptake value ratio approach, whole-brain and hippocampal volumes quantified from 3D-T1 MRI, and a composite cognitive score—the Preclinical Alzheimer Cognitive Composite (PACC). We investigated associations between blood pressure and blood pressure changes at and between 36, 43, 53, 60–64, and 69 years of age with WMHV using generalised linear models with a gamma distribution and log link function, amyloid-β status using logistic regression, whole-brain volume and hippocampal volumes using linear regression, and PACC score using linear regression, with adjustment for potential confounders.

**Findings:**

Between May 28, 2015, and Jan 10, 2018, 502 individuals were assessed as part of Insight 46. 465 participants (238 [51%] men; mean age 70·7 years [SD 0·7]; 83 [18%] amyloid-β-positive) were included in imaging analyses. Higher systolic blood pressure (SBP) and diastolic blood pressure (DBP) at age 53 years and greater increases in SBP and DBP between 43 and 53 years were positively associated with WMHV at 69–71 years of age (increase in mean WMHV per 10 mm Hg greater SBP 7%, 95% CI 1–14, p=0·024; increase in mean WMHV per 10 mm Hg greater DBP 15%, 4–27, p=0·0057; increase in mean WMHV per one SD change in SBP 15%, 3–29, p=0·012; increase in mean WMHV per 1 SD change in DBP 15%, 3–30, p=0·017). Higher DBP at 43 years of age was associated with smaller whole-brain volume at 69–71 years of age (−6·9 mL per 10 mm Hg greater DBP, −11·9 to −1·9, p=0·0068), as were greater increases in DBP between 36 and 43 years of age (−6·5 mL per 1 SD change, −11·1 to −1·9, p=0·0054). Greater increases in SBP between 36 and 43 years of age were associated with smaller hippocampal volumes at 69–71 years of age (−0·03 mL per 1 SD change, −0·06 to −0·001, p=0·043). Neither absolute blood pressure nor change in blood pressure predicted amyloid-β status or PACC score at 69–71 years of age.

**Interpretation:**

High and increasing blood pressure from early adulthood into midlife seems to be associated with increased WMHV and smaller brain volumes at 69–71 years of age. We found no evidence that blood pressure affected cognition or cerebral amyloid-β load at this age. Blood pressure monitoring and interventions might need to start around 40 years of age to maximise late-life brain health.

**Funding:**

Alzheimer's Research UK, Medical Research Council, Dementias Platform UK, Wellcome Trust, Brain Research UK, Wolfson Foundation, Weston Brain Institute, Avid Radiopharmaceuticals.

**Research in context**

**Evidence before this study**

Hypertension, particularly in midlife, is associated with late-life dementia risk and with risk of clinically diagnosed Alzheimer's disease. We reviewed previous work on blood pressure and brain health with a focus on white matter hyperintensities, brain volumes, and amyloid pathology. We searched PubMed for all articles published from database inception to Sept 31, 2018, with no language restrictions, for the keywords “blood pressure”, “hypertension”, “brain volume”, “hippocampal volume”, “atrophy”, “white matter hyperintensity”, “cerebral small vessel disease”, “amyloid”, “MRI”, “PET”, and “midlife”. Extensive published literature describes the positive association between hypertension and cerebral small vessel disease. Hypertension has also been associated with smaller brain volumes, with more consistent associations seen with midlife, rather than late life, measures of blood pressure. The association between blood pressure and amyloid burden is less clear. Several small studies found positive cross-sectional associations between higher blood pressure and elevated amyloid burden, whereas two large cohort studies, the Atherosclerosis Risk in Communities and the Mayo Clinic Study of Aging, did not observe an association between midlife hypertension and late-life amyloid burden. Midlife is generally defined as spanning a period between 40 years of age and 65 years of age. Studies to date have primarily investigated associations using a single measure in midlife, with the wide age range at measurement reducing the ability of these studies to determine whether there are specific periods in life when blood pressure, or change in blood pressure, particularly affect late-life brain health. Furthermore, some studies have used retrospective, clinically collected information on hypertension diagnosis, rather than prospectively collected blood pressure measurements from a community-based sample.

**Added value of this study**

Our study presents the first work, to our knowledge, examining the association between blood pressure taken prospectively at multiple timepoints, blood pressure changes, and brain pathologies and volumes measured systematically in a birth cohort in which all participants are of a near-identical age. The study design provides a unique opportunity to examine blood pressure changes in well-defined periods from early adulthood into midlife to late life and to explore their effect on brain pathologies and brain volumes. The use of data from this cohort, whose participants were assessed at the last visit over a 2-year period, enables examination of associations without the confounding effect of age, which is strongly associated with all these measures. We find that early midlife, perhaps extending back into early adulthood, is a particularly sensitive period when elevated blood pressure and rapid blood pressure increases are associated with the development of subsequent white matter hyperintensities and smaller brain volumes. We did not observe an association between blood pressure or blood pressure change and late-life amyloid burden, providing additional evidence that associations observed between midlife hypertension and late-life dementia risk are unlikely to be mediated through amyloidogenic pathways. At the age of 69–71 years, in this cognitively normal cohort, associations between life course measures of blood pressure and cognition were not detected.

**Implications of all the available evidence**

Our results build upon existing evidence regarding the role of blood pressure in the development of subsequent brain pathology. However, although raised blood pressure might be associated with dementia and clinically diagnosed Alzheimer's disease, it does not appear to affect risk for Alzheimer's disease through amyloidogenic pathways at 69–71 years of age. Based on our results, the fourth to sixth decades of life might be a sensitive period when changes in blood pressure are particularly damaging to the brain. Findings suggest that routine and serial blood pressure measurement might need to start earlier than is typically considered (eg, around 40 years of age), and decisions to start treatment might need to be based not only on absolute blood pressure, but also longitudinal blood pressure change.

## Introduction

Dementia affects 44 million people worldwide, a number predicted to triple by 2050.^1^ Up to a third of dementia risk might be modifiable, and a 5-year delay in onset could halve prevalence and associated costs.^2^ Hypertension is a well-established risk for vascular cognitive impairment mediated through cerebrovascular disease and has also been linked with Alzheimer's disease.^3^ Hypertension in midlife confers the greatest risk for cognitive impairment.^4^, ^5^ However, midlife spans a period of more than 20 years (age 40–65 years), and it is unknown whether a sensitive period exists in which elevated or changing blood pressure is particularly damaging; and if so, which pathological mechanisms are involved.

We sought to determine the association between blood pressure and longitudinal blood pressure changes across a period of about 33 years, spanning early adulthood (36 years), midlife (43–64 years), and early late life (69 years), and cerebral small vessel disease, brain volumes, and amyloid pathology in early late life. We hypothesised that blood pressure would be differentially related to markers of different brain pathologies, and that blood pressure and changes in blood pressure in specific periods would affect brain pathology.

## Methods

### Study design and participants

Participants were from Insight 46, a substudy of the Medical Research Council (MRC) National Survey of Health and Development (NSHD), a birth cohort that initially comprised 5362 individuals born throughout mainland Britain in 1 week in 1946,^6^ with follow-up including more than 20 contacts since birth. Eligibility criteria for Insight 46 have been described elsewhere (appendix p 1).^7^ Participants were assessed at University College London. Assessments included clinical and cognitive assessment and combined PET-MRI.^7^ A comparison of health and demographic characteristics between participants in Insight 46 and the larger MRC NSHD has previously been reported.^8^

Ethical approvals for the wider NSHD have been described.^9^ Insight 46 was approved by the Queen Square Research Ethics Committee. All participants provided written informed consent.

### Procedures

Imaging was done using a single Biograph mMR 3T PET-MRI scanner (Siemens Healthcare, Erlangen), with simultaneous acquisition of dynamic PET-MRI data, including volumetric (1·1 mm isotropic) T1-weighted and T2-weighted Fluid Attenuated Inversion Recovery (FLAIR) sequences. The full imaging protocol has been described previously.^7^ PET data were acquired continuously in list mode, during and following injection of 370 MBq ^18^F florbetapir (Avid Radiopharmaceuticals, Philadelphia, PA). Amyloid burden was assessed over a 10-min period, around 50 min after injection. Global standardised uptake value ratio was calculated from cortical regions of interest, normalised to eroded subcortical white matter (appendix p 1). Positive or negative amyloid status was determined using a Gaussian mixture model applied to standardised uptake value ratio values, taking the 99th percentile of the lower Gaussian as the cutoff (0·6104).

Volumetric T1-weighted and FLAIR images underwent visual quality control before processing using automated pipelines:^7^ whole-brain volume segmentation using Multi-Atlas Propagation and Segmentation,^10^ hippocampal volume using Similarity and Truth Estimation for Propagated Segmentations,^11^ with appropriate manual editing, and total intracranial volume using Statistical Parametric Mapping 12.^12^ A validated, unsupervised, automated algorithm, Bayesian Model Selection (BaMoS),^13^ was used to segment white matter hyperintensities jointly from 3D T1 and FLAIR images, followed by visual quality control, generating a global white matter hyperintensity volume (WMHV) including subcortical grey matter but excluding infratentorial regions. Further detail on BaMoS is provided in the appendix.

Neuropsychology included tests of episodic memory (Wechsler memory scale-revised logical memory test,^14^ Face-Name Associative Memory Exam 12^15^), processing speed (Wechsler adult intelligence scale-revised digit symbol substitution test^16^) and global cognition (Mini-Mental State Examination^17^), from which the Preclinical Alzheimer Cognitive Composite (PACC) was derived (appendix p 1).

Seated blood pressure was measured in the upper arm twice after 5 min of rest at ages 36, 43, 53, 60–64, and 69 years. At ages 36 and 43 years, a Hawksley Random Zero sphygmomanometer was used and, at ages 53, 60–64, and 69 years, an Omron HEM-705 automated digital oscillometric sphygmomanometer was used. To ensure compatibility, published conversion equations were used.^18^ The second blood pressure measure was used for analyses, unless missing. Hypertension was defined for descriptive purposes as blood pressure of 140/90 mm Hg or higher or self-reported clinical diagnosis. Prescription medication use was self-reported. At each timepoint, individuals were categorised according to use of antihypertensive medication.

Systolic blood pressure (SBP) and diastolic blood pressure (DBP) change for the periods between 36 and 43, 43 and 53, 53 and 60–64, and 60–64 and 69 years of age, conditional on earlier measurements, was calculated as the residual from the regression of each blood pressure measure (from 43 years of age) on the earlier measures for each sex, using individuals with available data at all time-points. Residuals represent changes in blood pressure that differed from changes expected on average given the earlier blood pressure. Residuals were standardised, allowing comparison between periods.^19^

Vascular risk factors used in fully adjusted models included smoking status, hypercholesterolaemia, diabetes, and body-mass index (BMI) at time of scanning (appendix p 1). Genotyping of the two single nucleotide polymorphisms, rs439358 and rs7412, determined *APOE* genotype. Individuals were categorised as *APOE* ɛ4 carriers or non-carriers. Adult socioeconomic position was defined as non-manual or manual on the basis of occupation at 53 years of age according to the UK Registrar General's Classification of Occupations. A childhood cognition score was calculated as described previously.^20^

### Statistical analysis

Analyses were done in Stata version 14.1. Participants were included if they were dementia-free based on expert consensus, informed by clinical history, informant history, and Mini-Mental State Examination score (≥26). For imaging analyses, participants also needed acceptable quality amyloid PET-MRI. For WMHV and brain volume analyses, individuals with white matter pathologies not considered to be of vascular origin (eg, demyelination) or cortical infarcts inappropriately segmented were excluded. For brain volume analysis, individuals also needed a useable amyloid scan. Otherwise, all participants with available blood pressure measures at any time-point were included in the analysis.

Blood pressure at each visit was compared between those in Insight 46 and the whole cohort, using an unadjusted linear mixed effect model for men and women separately, using all available blood pressure measurements. An unstructured residual variance-covariance matrix was used to model correlation between repeated measures in an individual.

Owing to the skewed distribution of WMHV, generalised linear models using the gamma distribution with log link were used to investigate associations between blood pressure at each age separately and WMHV at 69–71 years of age. The association between blood pressure and severe WMHV (top quintile of WMHV) was also investigated using logistic regression. Linear regression was used to investigate associations between blood pressure and whole-brain volume and mean hippocampal volumes at 69–71 years of age. Model 1 was adjusted for sex, total intracranial volume, and age-at-scan. Model 2 was also adjusted for antihypertensive medication use at time of blood pressure measurement. Model 3 additionally adjusted for blood pressure at 69 years of age to explore associations independently of current blood pressure. Model 4 additionally adjusted for potential cardiovascular confounders: smoking status, BMI, diabetic status, hypercholesterolaemia status, childhood cognition, and adult socioeconomic position. For brain volume analyses, to explore blood pressure influences independently of measurable brain pathologies, model 4 also adjusted for global WMHV and amyloid status.

Blood pressure change variables were then treated as the main predictor within generalised linear models or linear regression models. In model 1(c), all SBP or DBP conditional change variables were included and adjusted for sex, total intracranial volume and age-at-scan. In model 2(c), each conditional change variable was assessed individually and adjusted for change in antihypertensive medication status between timepoints under investigation and for other covariates described for model 4.

We used logistic regression to test associations between blood pressure at 36–69 years of age and amyloid status at age 69–71 years of age. Model 1 adjusted for sex. Model 2 further adjusted for *APOE* ɛ4 status. Model 3 additionally adjusted for antihypertensive medication use. Model 4 also adjusted for blood pressure (at 69 years of age). Additional vascular risk factors were not included in models due to the limited number of amyloid-positive individuals. Associations between blood pressure change and amyloid status were investigated using three models. Model 1(c) included all conditional change variables (SBP or DBP) within the same model, adjusting for sex. Model 2(c) additionally adjusted for *APOE* ɛ4 status. Model 3(c) assessed each trajectory separately and adjusted for change in antihypertensive medication status. A differential influence of blood pressure (or change) on amyloid status by *APOE* ɛ4 status was tested using an interaction term in fully adjusted models.

An exploratory analysis investigated associations between blood pressure and blood pressure change and cognition at 69–71 years of age, using the PACC. The linear regression models that we used are described in the appendix (p 2).

Model assumptions were checked with regression diagnostics, including checks of linearity by examination of residuals. Possible non-linear associations were explored by introducing a quadratic term to fully adjusted models. Any suggestion of a non-linear association (p<0·05) was investigated using linear spline modelling, with introduction of knot points, using standard cutoffs for (pre-)hypertension and hypotension, where appropriate. Statistical significance was set at p<0·05.

### Role of the funding source

The funders of the study had no role in study design, data collection, analysis, or interpretation, or report writing. All authors had full access to all the data in the study. The corresponding author had final responsibility for the decision to submit for publication.

## Results

Between May 28, 2015, and Jan 10, 2018, 502 participants were assessed as part of Insight 46 at University College London (appendix p 3). Of the individuals assessed, 471 (94%) completed the imaging protocol, of whom 468 (93%) were dementia-free. Age at Insight 46 assessment was similar in all individuals (mean 70·7 years [SD 0·7]). 83 (18%) were amyloid positive. Following imaging processing and quality control, data from 457 (91%) were available for amyloid analysis, 445 (89%) for brain volume analysis, and 453 (90%) for WMHV analysis. 499 dementia-free individuals were included in the cognitive analysis. Participant characteristics are summarised in table 1. Compared with those not completing the imaging protocol (n=31), participants with full imaging and blood pressure data available (n=398) had marginally lower blood pressure at 69 years of age (132·1/73·3 mm Hg *vs* 133·6/74·2 mm Hg), lower BMI (27·6 mm Hg *vs* 30·4 kg/m^2^), and lower prevalence of current smoking (3% *vs* 7%), diabetes (10% *vs* 17%), and hypercholesterolaemia (79% *vs* 87%; appendix p 4). Women in Insight 46 on average had SBP 1·8 mm Hg and DBP 0·9 mm Hg lower than those in the larger MRC NSHD cohort. Men in Insight 46 on average had SBP 2·1 mm Hg and DBP 0·6 mm Hg lower than those in the larger MRC NSHD cohort (appendix p 5).

**Table 1:** Characteristics of dementia-free participants with at least one outcome of interest

		**Participants assessed**			**Overall value**
		Men	Women	Overall	
Age at assessment		255	244	499	70·7 (0·7)
Age at scanning		238	227	465	70·7 (0·7)
Amyloid positive		232	225	457	83 (18%)
Whole brain volume, mL		227	218	445	1099·0 (98·4)
Hippocampal volume, mL		227	218	445	3·1 (0·3)
White matter hyperintensity volume, mL		233	220	453	3·1 (1·6–6·8)
Total intracranial volume, mL		233	220	453	1434·0 (132·3)
Mini-mental state examination score, out of 30		255	244	499	29·3 (0·9)
Preclinical Alzheimer Cognitive Composite z score		255	244	499	0·018 (0·66)
Standardised childhood cognition score		255	244	499	0·45 (0·64)
Systolic blood pressure, mm Hg					
	At 36 years of age	233	224	457	120·2 (13·7)
	At 43 years of age	243	232	475	123·5 (13·7)
	At 53 years of age	247	238	485	133·5 (19·0)
	At 60–64 years of age	255	243	498	134·9 (16·9)
	At 69 years of age	253	239	492	132·3 (16·1)
Diastolic blood pressure, mm Hg					
	At 36 years of age	233	224	457	78·4 (9·5)
	At 43 years of age	243	232	475	80·4 (9·3)
	At 53 years of age	247	238	485	83·1 (11·8)
	At 60–64 years of age	255	243	498	77·0 (9·4)
	At 69 years of age	253	239	492	73·4 (10·1)
Hypertensive*					
	At 36 years of age	231	224	455	71 (16%)
	At 43 years of age	243	232	475	104 (22%)
	At 53 years of age	247	241	488	226 (46%)
	At 60–64 years of age	255	244	499	259 (52%)
	At 69 years of age	253	241	494	274 (55%)
Antihypertensive medication use					
	At 36 years of age	234	226	460	7 (2%)
	At 43 years of age	247	235	482	9 (2%)
	At 53 years of age	248	242	490	57 (12%)
	At 60–64 years of age	255	244	499	141 (28%)
	At 69 years of age	251	235	486	194 (40%)
Smoking status at 68 years of age		255	244	499	..
	Current smoker	..	..	18	18 (4%)
	Ex-smoker	..	..	311	311 (62%)
	Never smoked	..	..	170	170 (34%)
Hypercholesterolaemia at 70 years of age†		255	244	499	398 (80%)
Diabetes at 70 years of age‡		254	240	494	55 (11%)
Body-mass index at 70 years of age, kg/m^2^		255	244	499	27·7 (4·5)
Adult socioeconomic position§		255	244	499	..
	Non-manual (Class I–IIIN)	..	..	424	424 (85%)
	Manual (Class IIIM–V)	..	..	75	75 (15%)
*APOE* ɛ4 carrier (1 or 2 alleles)		253	244	497	146 (29%)

Values are n, mean (SD), n (%), or median (IQR).

* Defined as blood pressure of 140/90 mm Hg or higher or self-reported clinical diagnosis.

† Determined on the basis of self-reported use of cholesterol-lowering medication at 70 years of age or random total cholesterol 5 mmol/L or more at 69 years of age.

‡ Determined on the basis of self-reported diabetic medication use at 70 years of age or HbA1c more than 6·5% or a self-reported diagnosis at 69 years of age.

§ Based on occupation at 53 years of age according to the UK Registrar General's Classification of Occupations.

Greater SBP and DBP at all timepoints was associated with greater WMHV, but this association only reached significance at 53 years of age (table 2; appendix p 7). In the fully adjusted model (model 4), a 10 mm Hg greater SBP at 53 years of age was associated with a relative increase in mean WMHV of 7% (relative increase 1·07, 95% CI 1·01–1·14, p=0·024), and a 10 mm Hg greater DBP was associated with a 15% increase in mean WMHV (1·15, 1·04–1·27, p=0·0057). A similar association was seen between blood pressure at 53 years of age and severe WMHV (appendix p8). Introduction of a quadratic term suggested a non-linear association between DBP at 36 years of age and WMHV (quadratic p=0·015). A spline model with knot points at 60 mm Hg and 90 mm Hg found no association at blood pressures less than 90 mm Hg but a significant positive association at blood pressures more than 90 mm Hg (exponentiated coefficient 2·07, 95% CI 1·13–3·79, p=0·018; appendix p 13). No non-linear associations were observed at other timepoints. Greater increases in SBP and DBP between timepoints seemed to be associated with increased WMHV, with a significant association at 43–53 years of age (appendix pp 6–7), with no evidence of non-linear associations (p>0·17, all tests). A 1 SD greater increase in SBP at 43–53 years of age was associated with 15% larger WMHV (1·15, 95% CI 1·03–1·29, p=0·012), and one SD greater increase in DBP was associated with 15% larger WMHV (1·15, 1·03–1·20, p=0·017; appendix p 6).

**Table 2:** Associations between blood pressure (per 10 mm Hg increase) and global white matter hyperintensity volume at 69–71 years of age

	**Model 1**			**Model 2**			**Model 3**			**Model 4**		
	n	Relative increase in WMHV (95% CI)	p value	n	Relative increase in WMHV (95% CI)	p value	n	Relative increase in WMHV (95% CI)	p value	n	Relative increase in WMHV (95% CI)	p value
**Systolic blood pressure**												
36 years of age	413	1·02 (0·94–1·10)	0·64	412	1·01 (0·93–1·09)	0·87	408	0·99 (0·92–1·07)	0·89	408	1·00 (0·92–1·08)	0·95
43 years of age	430	1·05 (0·98–1·14)	0·17	430	1·05 (0·98–1·14)	0·18	424	1·04 (0·97–1·12)	0·27	424	1·04 (0·97–1·13)	0·27
53 years of age	441	1·10 (1·04–1·16)	0·0010	441	1·09 (1·03–1·15)	0·0018	435	1·08 (1·02–1·14)	0·011	435	1·07 (1·01–1·14)	0·024
60–64 years of age	452	1·05 (0·99–1·12)	0·094	452	1·05 (0·98–1·11)	0·16	446	1·03 (0·96–1·10)	0·43	446	1·02 (0·95–1·09)	0·57
69 years of age	447	1·07 (1·00–1·14)	0·047	441	1·06 (1·00–1·13)	0·053	NA	NA	NA	441	1·06 (1·00–1·13)	0·067
**Diastolic blood pressure**												
36 years of age	413	1·08 (0·97–1·19)	0·16	412	1·06 (0·96–1·18)	0·26	408	1·06 (0·95–1·18)	0·31	408	1·06 (0·96–1·18)	0·25
43 years of age	430	1·06 (0·95–1·19)	0·28	430	1·06 (0·95–1·19)	0·29	424	1·05 (0·94–1·17)	0·41	424	1·05 (0·94–1·18)	0·38
53 years of age	441	1·17 (1·07–1·28)	0·0006	441	1·17 (1·06–1·28)	0·0009	435	1·16 (1·05–1·27)	0·0029	435	1·15 (1·04–1·27)	0·0057
60–64 years of age	452	1·12 (1·00–1·25)	0·054	452	1·11 (0·99–1·24)	0·078	446	1·09 (0·96–1·23)	0·17	446	1·08 (0·96–1·23)	0·21
69 years of age	447	1·07 (0·97–1·18)	0·20	441	1·07 (0·97–1·19)	0·18	NA	NA	NA	441	1·07 (0·97–1·19)	0·19

Model 1 was adjusted for sex, total intracranial volume, and age at MRI scan. Model 2 was identical to model 1 plus adjusted for antihypertensive medication at given timepoint. Model 3 was identical to model 2 plus adjusted for blood pressure at 69 years of age. Model 4 was identical to model 3 plus adjusted for adult socioeconomic position, childhood cognition, smoking status, cholesterol status, diabetes, and body-mass index. WMHV=White matter hyperintensity volume. NA=not applicable. For ease of interpretation, we exponentiated the coefficients to give the proportional effect of a 10 mm Hg greater blood pressure on mean WMHV. For example, an exponentiated coefficient of 1·1 represents a 10% increase in mean WMHV.

There was no evidence that blood pressure at any age was associated with amyloid status (table 3). Blood pressure changes were not associated with amyloid status (appendix p 9). *APOE* ɛ4 status did not affect associations between blood pressure or blood pressure change and amyloid status (interaction p values all >0·13). There was no evidence of non-linear associations (p>0·13, all tests).

**Table 3:** Associations between blood pressure (per 10 mm Hg increase) and amyloid status at 69–71 years of age

	**Model 1**			**Model 2**			**Model 3**			**Model 4**		
	n	Adjusted odds ratio (95% CI)	p value	n	Adjusted odds ratio (95% CI)	p value	n	Adjusted odds ratio (95% CI)	p value	n	Adjusted odds ratio (95% CI)	p value
**Systolic blood pressure**												
36 years of age	416	1·01 (0·83–1·22)	0·96	414	1·06 (0·87–1·30)	0·55	413	1·07 (0·87–1·31)	0·52	409	1·07 (0·87–1·32)	0·52
43 years of age	434	0·96 (0·79–1·16)	0·67	432	0·99 (0·81–1·20)	0·90	432	0·99 (0·81–1·20)	0·88	426	0·98 (0·80–1·20)	0·84
53 years of age	444	0·95 (0·83–1·08)	0·40	442	0·91 (0·79–1·04)	0·17	442	0·91 (0·79–1·05)	0·18	436	0·89 (0·76–1·04)	0·14
60–64 years of age	456	0·98 (0·85–1·13)	0·80	454	0·94 (0·81–1·09)	0·41	454	0·95 (0·82–1·11)	0·54	448	0·93 (0·78–1·11)	0·42
69 years of age	451	1·05 (0·90–1·22)	0·54	449	1·01 (0·86–1·18)	0·92	443	1·02 (0·87–1·20)	0·83	NA	NA	NA
**Diastolic blood pressure**												
36 years of age	416	0·90 (0·69–1·17)	0·43	414	0·91 (0·69–1·21)	0·52	413	0·92 (0·69–1·22)	0·55	409	0·93 (0·70–1·24)	0·61
43 years of age	434	0·91 (0·69–1·21)	0·53	432	0·91 (0·67–1·21)	0·51	432	0·91 (0·68–1·22)	0·52	426	0·92 (0·68–1·24)	0·59
53 years of age	444	0·97 (0·79–1·20)	0·80	442	0·92 (0·73–1·16)	0·48	442	0·92 (0·73–1·16)	0·50	436	0·93 (0·73–1·17)	0·51
60–64 years of age	456	0·96 (0·75–1·25)	0·79	454	0·92 (0·70–1·21)	0·53	454	0·93 (0·70–1·23)	0·62	448	0·92 (0·69–1·25)	0·61
69 years of age	451	1·04 (0·82–1·32)	0·73	449	0·97 (0·76–1·25)	0·83	443	0·96 (0·75–1·24)	0·77	NA	NA	NA

Model 1 was adjusted for sex. Model 2 was identical to model 1 plus adjusted for *APOE* ɛ4 status. Model 3 was identical to model 2 plus adjusted for concurrent antihypertensive medication. Model 4 was identical to model 3 plus adjusted for blood pressure at 69 years of age. NA=not applicable.

There was some evidence to suggest that higher SBP across timepoints might be associated with smaller whole-brain volume, with significant effects at 43 years of age (model 3, p=0·041) and 53 years of age (model 3, p=0·011; table 4). Although associations were attenuated in fully adjusted models, they remained significant at 53 years of age (model 4, p=0·039; table 4). DBP at 43 years of age had a negative effect on whole-brain volume, including in the fully adjusted model (model 4, p=0·0068; table 4; appendix pp 12); a 10 mm Hg higher DBP at that age was associated with a 6·9 mL reduction (95% CI −11·9 to −1·9) in whole-brain volume. Higher SBP at 43 years of age had a similar association with smaller mean hippocampal volume across models (model 4, p=0·048; table 5). In the fully adjusted model, a 10 mm Hg higher SBP was associated with a 0·021 mL (95% CI −0·043 to −0·0002) smaller hippocampal volume. We found no associations between DBP and hippocampal volume (table 5).

**Table 4:** Associations between blood pressure (per 10 mm Hg increase) and whole brain volume at 69–71 years of age

	**Model 1**			**Model 2**			**Model 3**			**Model 4**		
	n	β (95% CI)	p value	n	β (95% CI)	p value	n	β (95% CI)	p value	n	β (95% CI)	p value
**Systolic blood pressure**												
36 years of age	405	−2·3 (−5·8 to 1·2)	0·20	404	−2·3 (−5·8 to 1·2)	0·20	400	−2·2 (−5·8 to 1·4)	0·23	400	−2·2 (−5·7 to 1·3)	0·21
43 years of age	422	−3·9 (−7·2 to −0·6)	0·021	422	−3·9 (−7·2 to −0·6)	0·021	416	−3·5 (−6·9 to −0·1)	0·041	416	−3·0 (−6·4 to 0·3)	0·077
53 years of age	433	−3·0 (−5·3 to −0·8)	0·0084	433	−3·2 (−5·5 to −1.0)	0·0056	427	−3·3 (−5·8 to −0·7)	0·011	427	−2·7 (−5·2 to −0·1)	0·039
60–64 years of age	444	−1·8 (−4·3 to 0·7)	0·16	444	−1·5 (−4·0 to 1·0)	0·24	438	−0·9 (−3·7 to 1·9)	0·52	438	−0·4 (−3·2 to 2·5)	0·81
69 years of age	439	−1·8 (−4·4 to 0·8)	0·18	433	−1·7 (−4·4 to 0·9)	0·20	NA	NA	NA	433	−2·1 (−4·7 to 0·6)	0·12
**Diastolic blood pressure**												
36 years of age	405	−0·5 (−5·3 to 4·2)	0·82	404	−0·5 (−5·3 to 4·3)	0·85	400	−0·6 (−5·5 to 4·4)	0·82	400	0·3 (−4·5 to 5·2)	0·90
43 years of age	422	−6·6 (−11·5 to −1·7)	0·0082	422	−6·7 (−11·6 to −1·8)	0·0080	416	−7·0 (−12·0 to −1·9)	0·0072	416	−6·9 (−11·9 to −1·9)	0·0068
53 years of age	433	−2·5 (−6·3 to 1·3)	0·20	433	−2·6 (−6·5 to 1·2)	0·18	427	−2·8 (−6·9 to 1·2)	0·17	427	−2·0 (−6·2 to 2·1)	0·34
60–64 years of age	444	−0·8 (−5·3 to 3·7)	0·71	444	−0·4 (−4·9 to 4·1)	0·85	438	−0·3 (−5·2 to 4·7)	0·92	438	0·4 (−4·6 to 5·4)	0·87
69 years of age	439	−0·8 (−5·0 to 3·3)	0·70	433	−0·9 (−5·1 to 3·3)	0·68	NA	NA	NA	433	−1·8 (−6·1 to 2·5)	0·41

Model 1 was adjusted for sex, total intracranial volume, and age at MRI scan. Model 2 was identical to model 1 plus adjusted for antihypertensive medication at given age. Model 3 was identical to model 2 plus adjusted for blood pressure at 69 years of age. Model 4 was identical to model 3 plus adjusted for adult socioeconomic position, childhood cognition, smoking status, cholesterol status, diabetes, body-mass index, amyloid status, and white matter hyperintensity volume. NA=not applicable.

**Table 5:** Associations between blood pressure (per 10 mm Hg increase) and mean hippocampal volume at 69–71 years of age

	**Model 1**			**Model 2**			**Model 3**			**Model 4**		
	n	β (95% CI)	p value	n	β (95% CI)	p value	n	β (95% CI)	p value	n	β (95% CI)	p value
**Systolic blood pressure**												
36 years of age	405	−0·005 (−0·026 to 0·017)	0·68	404	−0·0033 (−0·025 to 0·019)	0·77	400	−0·004 (−0·027 to 0·018)	0·70	400	−0·005 (−0·028 to 0·017)	0·66
43 years of age	422	−0·022 (−0·043 to −0·001)	0·037	422	−0·022 (−0·042 to −0·001)	0·040	416	−0·022 (−0·043 to −0·001)	0·040	416	−0·021 (−0·043 to −0·0002)	0·048
53 years of age	433	−0·010 (−0·024 to 0·006)	0·22	433	−0·012 (−0·027 to 0·002)	0·10	427	−0·013 (−0·029 to 0·003)	0·12	427	−0·016 (−0·032 to 0·0003)	0·055
60–64 years of age	444	−0·010 (−0·026 to 0·006)	0·20	444	−0·012 (−0·028 to 0·004)	0·15	438	−0·011 (−0·029 to 0·007)	0·23	438	−0·013 (−0·031 to 0·006)	0·18
69 years of age	439	−0·007 (−0·023 to 0·010)	0·43	433	−0·008 (−0·025 to 0·009)	0·34	NA	NA	NA	433	−0·007 (−0·025 to 0·010)	0·39
**Diastolic blood pressure**												
36 years of age	405	0·001 (−0·029 to 0·031)	0·93	404	0·003 (−0·027 to 0·033)	0·83	400	−0·003 (−0·034 to 0·028)	0·85	400	−0·003 (−0·034 to 0·028)	0·85
43 years of age	422	−0·009 (−0·040 to 0·022)	0·56	422	−0·010 (−0·041 to 0·022)	0·55	416	−0·018 (−0·050 to 0·014)	0·27	416	−0·019 (−0·051 to 0·013)	0·25
53 years of age	433	−0·005 (−0·029 to 0·020)	0·70	433	−0·008 (−0·032 to 0·017)	0·54	427	−0·011 (−0·037 to 0·015)	0·39	427	−0·015 (−0·042 to 0·011)	0·26
60–64 years of age	444	0·019 (−0·010 to 0·048)	0·20	444	0·018 (−0·011 to 0·047)	0·23	438	0·017 (−0·015 to 0·049)	0·29	438	0·016 (−0·016 to 0·048)	0·33
69 years of age	439	0·006 (−0·020 to 0·033)	0·64	433	0·005 (−0·023 to 0·032)	0·74	NA	NA	NA	433	0·005 (−0·022 to 0·033)	0·70

Model 1 was adjusted for sex, total intracranial volume, and age at MRI scan. Model 2 was identical to model 1 plus adjusted for antihypertensive medication at given age. Model 3 was identical to model 2 plus adjusted for blood pressure at 69 years of age. Model 4 was identical to model 3 plus adjusted for adult socioeconomic position, childhood cognition, smoking status, cholesterol status, diabetes, body-mass index, amyloid status, and white matter hyperintensity volume. NA=not applicable.

At 53 years of age, there was evidence of a non-linear association between SBP and whole-brain volume (quadratic p=0·011) and between SBP and hippocampal volume (quadratic p=0·044). Using knot points at 120 mm Hg and 140 mm Hg and a fully adjusted model, no association existed below 140 mm Hg, but a negative association did exist between greater SBP and whole-brain volume at blood pressures greater than 140 mm Hg (β coefficient −9·0, 95% CI −14·3 to −3·8, p=0·0008; appendix pp 12–13), suggesting that 140 mm Hg is a crucial threshold above which SBP at 53 years of age adversely affects whole-brain volume in late life. A similar non-linear pattern was observed for hippocampal volume (SBP >140 mm Hg β-coefficient −0·05, 95% CI −0·08 to −0·01, p=0·0063). Introduction of an additional knot point at 130 mm Hg did not alter findings (appendix p 13).

Greater increases in SBP between 36 and 43 years of age showed evidence of a borderline linear association, and greater increases between 43 and 53 years of age showed a significant linear association with smaller whole-brain volume. These associations were attenuated in the fully adjusted model (appendix p 10). Further analysis found that there were non-linear associations between SBP change variables at 36–43 years of age (quadratic p=0·034) and 43–53 years of age (quadratic p=0·0074). Linear spline modelling with knot point at the average expected change (residual=0) showed that, among individuals with greater than expected change (residual >0), a greater SBP increase between 36 and 43 years of age (β-coefficient −10·6, −18·6 to −2·7, p=0·0091) and 43 and 53 years of age (β-coefficient −12·0, −20·0 to −4·0, p=0·0033) was associated with smaller whole-brain volume. There was no evidence of an association in individuals with less than expected change in SBP (residual <0) at 36–43 years of age (p=0·18) or 43–53 years of age (p=0·11; appendix p 13). There was a negative linear association between DBP changes at 36–43 years of age and whole-brain volume, whereby a one SD greater increase in DBP at 36–43 years of age was associated with a 6·5 mL (95% CI −11·1 to −1·9) reduction in whole-brain volume (model 2, p=0·0054; appendix p 10).

Greater increases in SBP between 36 and 43 years of age were associated with smaller hippocampal volume. A one SD greater increase in SBP between 36 and 43 years was associated with 0·03 mL (95% CI −0·06 to −0·001) smaller hippocampal volume (model 2, p=0·043; appendix pp 11–12). There was no evidence of non-linear associations (p>0·13 all tests). DBP changes were not associated with hippocampal volume. All associations with hippocampal volume were substantially attenuated when whole-brain volume was accounted for (data not shown).

No consistent associations were found between SBP or DBP at any age or blood pressure change and performance on the PACC (appendix pp 14–15).

Observing that the greatest mean increase in SBP was at 43–53 years of age, and the greatest rate of decline in DBP was between 53 and 60–64 years of age (appendix p 5), we included SBP change at 43–53 years of age and DBP change between 53 and 60–64 years of age in a fully adjusted model, introducing an interaction term between the two change variables. This analysis suggested that there could be a greater effect of declining DBP on WMHV burden at 69–71 years of age in those who previously had a greater SBP increase (interaction p=0·038; appendix p 17–18). This interaction was not observed in amyloid (interaction p=0·83), brain volume (whole-brain volume interaction p=0·42; hippocampal volume interaction p=0·32), or cognitive (interaction p=0·32) models. To explore whether this finding might be driven by changes in arterial stiffness, we examined the association between pulse pressure change variables and WMHV. None of these associations were significant (data not shown).

In fully adjusted models including adjustment for total intracranial volume, men had less WMHV (p=0·0063) and smaller whole-brain volume (p=0·0032) than women. Diabetics had smaller whole-brain volume (p=0·0074; appendix p 16). *APOE* ɛ4 carriers were more likely to be amyloid positive (p<0·0001; appendix p 16). Male sex (p<0·0001) and lower socioeconomic position (p=0·0009) predicted worse cognition (appendix p 16). Childhood cognition was positively associated with the PACC (p<0·0001; appendix p 16). No consistent associations existed between antihypertensive medication use and outcome measures (appendix p 16).

## Discussion

In this cohort of dementia-free individuals of virtually identical age recruited from across mainland Britain, we show that, even accounting for blood pressure in late life, higher blood pressure in midlife seems to be associated with greater cerebral small vessel disease burden and smaller whole-brain and hippocampal volumes at around 70 years of age. We show associations between more rapid blood pressure increases in early midlife, and smaller brain volumes and greater WMHV in late life. We found no evidence that blood pressure or blood pressure changes affect amyloid status.

Our observations are in agreement with previous work showing a particularly strong association between midlife blood pressure and late-life cerebral small vessel disease.^21^ Both SBP and DBP at 53 years of age were associated with late-life cerebral small vessel disease, although the effect size was almost double for DBP than for SBP. We found some evidence of a non-linear association between DBP in early adulthood, at 36 years of age, and late-life WMHV, with DBP increases greater than 90 mm Hg associated with greater WMHV. However, we did not find non-linear associations at other timepoints, so this finding needs further confirmation.

Although several cross-sectional studies have reported an association between higher blood pressure and amyloid burden,^22^, ^23^ our negative finding is consistent with those from two large cohort studies that specifically examined midlife hypertension.^24^, ^25^ Our work extends these previous findings by using continuous measures of blood pressure, exploring the associations with SBP and DBP separately using prospectively acquired measures at multiple time-points, as well as specifically investigating blood pressure changes over time. We also found no evidence of interaction between *APOE* ɛ4 carrier status, blood pressure, or blood pressure changes and amyloid status at 69–71 years of age. Taken together, these data provide further evidence that, if hypertension increases Alzheimer's disease risk, this increase occurs through non-amyloidogenic pathways, at least in those affected by their early 80s.

Greater DBP at 43 years of age and SBP at 53 years of age were most associated with smaller whole-brain volume, independently of contemporaneous blood pressure. There was a non-linear association with SBP at 53 years of age, whereby measures higher than 140 mm Hg were particularly detrimental to late-life whole-brain volume, suggesting a threshold effect for SBP. Similarly, greater increases in DBP at 36–43 years of age and greater increases in SBP at 36–53 years of age (ie, from early adulthood into midlife) were both associated with smaller whole-brain volume, independent of white matter hyperintensity load and amyloid burden. These findings are consistent with evidence from the Framingham Heart Study that vascular risk factors at younger ages were most strongly associated with lower brain volume in later life.^26^ We did not observe disproportionate hippocampal volume loss, as would be typical for Alzheimer's disease, suggesting that raised blood pressure mediates its damaging effects through global brain volume changes. Potential mechanisms by which raised blood pressure might influence brain volume include tau pathology^27^ or hypertension-related microstructural damage or infarction.^28^ Finally, we cannot exclude the possibility that blood pressure and brain volume share common predictors (eg, genetic factors). Previous work in the NSHD cohort has shown associations between rapid blood pressure rises at 43–53 years of age and greater left cardiac ventricular mass at 60–64 years of age, suggesting that this period is sensitive for cardiac as well as cerebral health^19^ or that cardiac changes might be on the causal pathway to cerebral damage.

We did not observe an association between blood pressure and cognition, as reported in other cohorts.^29^, ^30^, ^31^ We used a composite cognitive measure that might not have been cross-sectionally sensitive, particularly in a dementia-free cohort, noting that previous studies^30^, ^31^ have found associations between blood pressure and longitudinal cognitive change. Although the PACC incorporates a measure of executive function shown to be the cognitive domain most strongly influenced by blood pressure,^30^ it was originally developed to be sensitive to cognitive changes in preclinical Alzheimer's disease (before the development of overt cognitive symptoms); therefore, our findings are consistent with the absence of association between blood pressure and amyloid status.

Previous studies have produced conflicting findings regarding whether elevated SBP or DBP more strongly affects cerebral small vessel disease and brain volumes.^32^, ^33^, ^34^ Differences might relate to the different populations studied, or timing of measurements, perhaps mediated by differential endothelial sensitivity to haemodynamic changes at different ages. As with other studies, we observed different patterns of change in SBP and DBP over time. DBP declines after 50–60 years of age, whereas SBP continues to increase.^35^ Hypotension might be more detrimental in late life to cerebral small vessel disease and brain volumes in cognitively normal individuals, particularly if previously hypertensive,^21^, ^36^ perhaps due to shifts in the autoregulatory curve and cerebral hypoperfusion. We found some evidence for this finding, showing a greater effect of declining DBP on WMHV burden, but not brain volume, in individuals who had previously had a greater rise in SBP. Alternatively, this finding might be mediated by increasing arterial stiffness, which is associated with declining DBP, although we did not find a direct association between pulse pressure, a proxy marker of arterial stiffness, and subsequent WMHV.

Our findings are particularly relevant considering the findings from the SPRINT MIND study,^37^ which showed that intensive blood pressure control reduced risk of mild cognitive impairment, but not dementia. This finding might be partly due to the early termination of the trial owing to clear cardiovascular benefits in the intensive treatment group. However, the older age of participants (2636 [28%] of 9361 aged ≥75 years) might also have contributed, and our data would suggest that, although vascular risk control is important at all ages, effects on blood pressure might be more pronounced if targeted at an earlier age.

This study has several strengths. Participants are broadly representative of the population of mainland Britain born in 1946 and were of similar age at assessment. All participants had serial prospective measurements of blood pressure, allowing dynamic changes in blood pressure across the life course to be studied. Limitations inherent in any birth cohort are secular effects. All individuals went through midlife at the same time and are likely to have been exposed to similar hypertension treatments and targets, which might differ from those of individuals born in other periods. Furthermore, although participants have similar blood pressure profiles to the main cohort, we have previously shown that individuals with lower socioeconomic position, lower cognition, and more health problems are under-represented in Insight 46, suggesting that we might underestimate effect sizes compared with the general population.^8^ Other limitations include the use of static rather than ambulatory blood pressure measurements, which more closely correlate with end-organ damage.^38^ We treated amyloid burden as a binary variable, which is a common approach, but means that small blood pressure influences on amyloid burden might not be detected. We did not investigate associations with lacunes or subcortical infarcts, noting that these were present in a small proportion (36 [7%] of 471), or microbleeds, and we do not have measures of tau pathology. Insight 46 is a cohort consisting of exclusively white British participants, which might reduce generalisability to non-white populations, and their relatively young age (early 70s) might reduce generalisability to older populations who have larger pathological burden. This cohort is mostly cognitively healthy, meaning that we can show associations between blood pressure and brain pathology, but not with cognitive impairment.

This work shows that the fourth to sixth decade, from early adulthood into midlife, might be a sensitive period when greater blood pressure affects brain pathologies in late life, and that greater increases in blood pressure and, in the case of DBP, decline thereafter might be particularly harmful. These findings suggest that routine serial blood pressure measurement might need to start around 40 years of age (if not before), decisions to start treatment should account for longitudinal blood pressure change, and different approaches to blood pressure management might be needed at different ages. Associations between blood pressure and increased risk for dementia are likely to be mediated through effects on cerebral small vessel disease and brain volumes and not amyloid accumulation.

## Data sharing

A data-sharing policy is available on the NSHD Data Sharing website.
