# Correction to *Lancet Neurol* 2019; 18: 942–52

**DOI:** 10.1016/S1474-4422(20)30190-3

**Published:** 2020-07

*Lane CA, Barnes J, Nicholas JM, et al. Associations between blood pressure across adulthood and late-life brain structure and pathology in the neuroscience substudy of the 1946 British birth cohort (Insight 46): an epidemiological study.* Lancet Neurol *2019; **18:** 942–52*—The appendix of this Article has been corrected as of June 17, 2020.

