# Supplementary material for: Associations between blood pressure across adulthood and late-life brain structure and pathology in the neuroscience substudy of the 1946 British birth cohort (Insight 46): an epidemiological study
Source: Lancet Neurol. 2019 Oct;18(10):942–52. doi: 10.1016/S1474-4422(19)30228-5 (PMC6744368; doi:10.1016/S1474-4422(19)30228-5)
Supplement: Supplementary appendix [file mmc1.pdf]

THE LANCET
Neurology

Supplementary appendix

This appendix formed part of the original submission and has been peer reviewed. We post it as supplied by the authors.

Supplement to: Lane CA, Barnes J, Nicholas JM, et al. Associations between blood pressure across adulthood and late-life brain structure and pathology in the neuroscience substudy of the 1946 British birth cohort (Insight 46): an epidemiological study. *Lancet Neurol* 2019; published online Aug 20. http://dx.doi.org/10.1016/S1474-4422(19)30228-5.

Associations between blood pressure across adulthood and late-life brain structure and pathology in the neuroscience substudy of the 1946 British birth cohort (Insight 46): an epidemiological study

Supplementary Appendix

Supplemental methods

To capitalise on the life course data and to avoid a priori decisions as to who might be at risk of cognitive decline, entry criteria for Insight 46 were based on maximising the life course data available for analysis. MRC NSHD study members were recruited from those who attended a clinic-based assessment age 60-64 years, had previously indicated willingness to attend a clinic visit in London and for whom relevant data in childhood and adulthood were available, including at least one measure of childhood cognition and at least one BP measurement from ages 36, 43, 53 and 60-64 years. Individuals were excluded if they had contraindications to MRI or PET including, but not limited to, claustrophobia, metallic implants such as pacemakers, or research scans within the last year that would result in an individual exceeding acceptable mandated yearly radiation exposures.[1]

Amyloid PET global SUVR calculation: GIF v.3[2] region of interests (ROIs) were selected to match as closely as possible the Freesurfer (version 4·5·0, surfer.nmr.mgh.harvard.edu/) composite ROI used in Alzheimer's Disease Neuroimaging Initiative (ADNI), which includes the lateral and medial frontal, anterior and posterior cingulate, lateral parietal, and lateral temporal regions.[3]

Bayesian Model Selection (BaMoS) white matter hyperintensity (WMH) segmentation: BaMoS, an unsupervised automated algorithm, is based on a novel adaptive framework for the modelling of data outliers using a multivariate Gaussian mixture model[4] that has been shown to perform well against other freely-available automated segmentation methods for segmentation of age-related WMH.[4] Volumetric FLAIR and T1-weighted images were used for analysis. Prior to WMH segmentation, scans were reviewed by a consultant neuroradiologist, and any potential reportable findings flagged for review, according to pre-defined and published criteria.[1] Pre-processing involved rigid registration of subjects' FLAIR image to the T1 volumetric space using the NiftyReg opensource package,[5] followed by brain masking. Intensity data were log transformed and corrected for intensity inhomogeneities using an additive model of bias field as a linear combination of spatially varying polynomial functions.[6] The data are modelled hierarchically by separating the data into an inlier and an outlier part. Each part is symmetrically and jointly modelled as a mixture of multiple anatomical classes, with each one of these classes modelled as a combination of Gaussian distributions. Because the number of Gaussians necessary to characterise each tissue class is not known *a priori*, a split and merge strategy dynamically modifies the model structure and enables a more comprehensive investigation of the data space. After convergence of each newly suggested model using an expectation-maximisation algorithm, the Bayesian Information Criterion (BIC) is used to decide whether to accept or reject this new stage. Such criterion enforces appropriate balance between model fit and complexity.[7] Spatial constraints are introduced through anatomical probabilistic atlases while smoothness of the segmentation is enforced by the application of a Markov Random Field.[4] After model convergence, a post-processing step was applied in order to select candidate lesion voxels based on intensity and location rules. The formed connected components of candidate lesions were then automatically classified as lesion or artefact. The resulting probability map of WMH was then integrated to obtain the global white matter hyperintensity volume (WMHV), which included subcortical grey matter but not the infratentorial region. Validation of the BaMoS algorithm in Insight 46 was performed by comparing its performance with semi-automated segmentations, performed by two trained raters (CL and JB) on 30 Insight 46 scans. Good spatial agreement was demonstrated with both raters' semi-automated segmentations (mean (SD) Dice coefficients 0.71 (0.11); 0.72 (0.11) respectively).

Preclinical Alzheimer Cognitive Composite (PACC) generation: The four components of our version of the PACC were: MMSE total score, Logical Memory delayed recall score, Digit-Symbol Substitution score and FNAME-12 total score. Following the method described in previous studies,[8,9] the four components were converted into z-scores and then averaged. A higher PACC score indicates better performance. Two participants did not complete the FNAME task. For these two participants, their PACC score was the average of the z-scores for the three tests they completed.

Vascular risk factor covariates: Smoking status was defined by questionnaire (at age 68 years, or if missing at 60-64 years) as: never-smoked, ex-smoker, current smoker. Hypercholesterolaemia status was determined on

self-reported use of cholesterol-lowering medication at 70 or random total cholesterol ≥ 5mmol/L at 69 years. Diabetes mellitus (DM) status was based on self-reported diabetic medication use at 70 years or HbA1c>6·5% or a self-reported diagnosis at 69 years. Body mass index (BMI) was defined as weight(kg)/height(m^2) at age 69-71 years.

Cognitive statistical analyses: Linear regression models were used to investigate the associations between BP and change in BP and cognition at age 69-71 years, using the PACC. Model 1 adjusted for sex, childhood cognition and age at assessment. Model 2 also adjusted for contemporaneous antihypertensive medication usage. Model 3 additionally adjusted for BP at age 69 years to explore relationships independently of 'current' BP. Model 4 additionally adjusted for other potential cardiovascular confounders: smoking status, BMI, diabetic status, hypercholesterolaemia status and adult SEP. Associations between BP change variables and PACC score were investigated using two models. Model 1(c): all SBP or DBP change variables included and adjusted for sex, childhood cognition and assessment age. Model 2(c): each change variable assessed individually and adjusted for change in antihypertensive medication status between the two time-points under investigation, and other covariates described in model 4 above.

Supplementary figure 1 Flowchart providing an overview of Insight 46 recruitment from the MRC NSHD (modified with permission from James *et al*[10] under the terms of the Creative Commons Attribution 4.0 International License (http://creativecommons.org/licenses/by/4.0/)) and summary of imaging data available. BaMoS, Bayesian Model Selection; MRI, magnetic resonance imaging; MCI, mild cognitive impairment; NSHD, National Survey of Health and Development; PET, positron emission tomography; QC, quality control; WMHV, white matter hyperintensity volume.

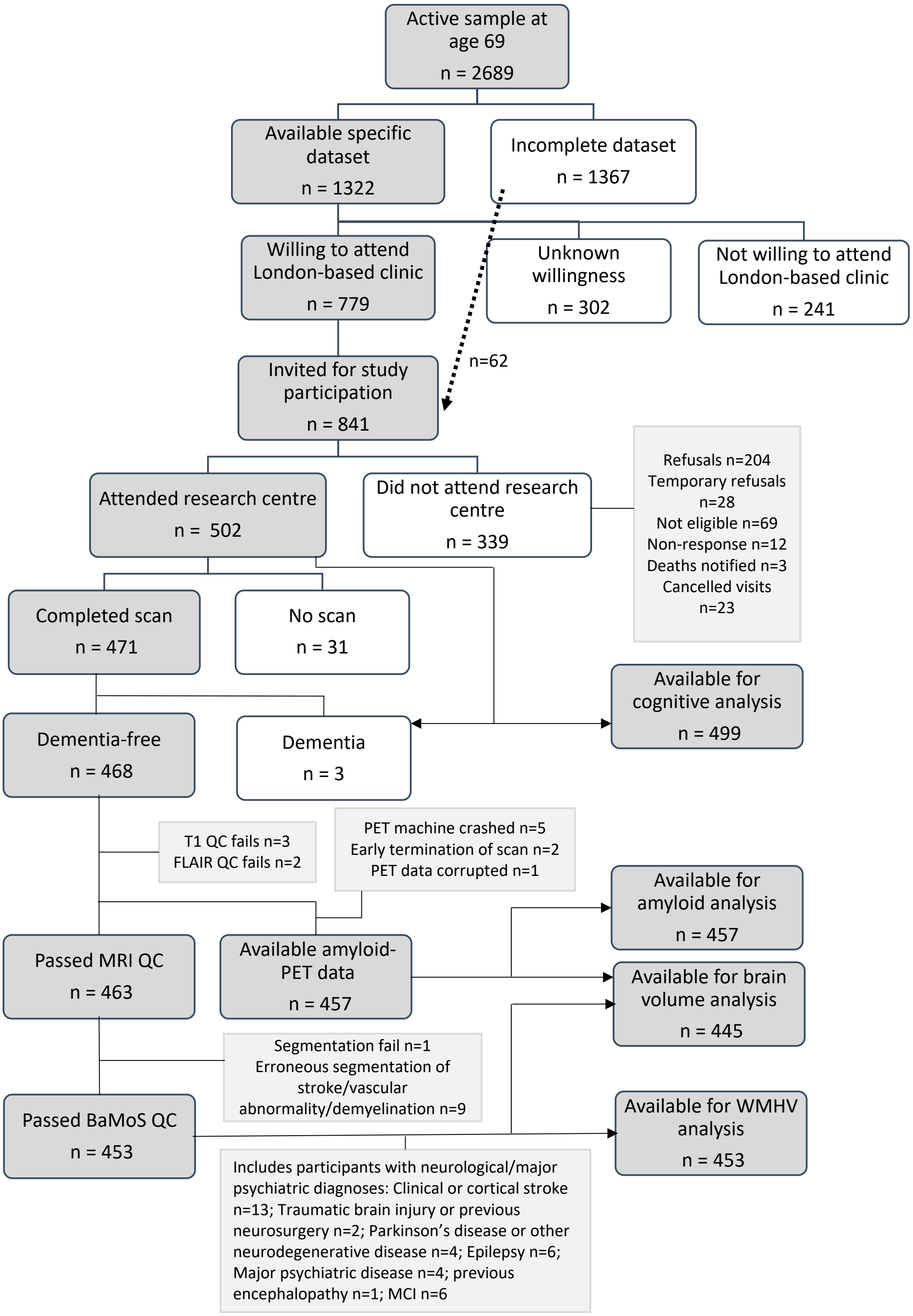

Active sample at age 69
n = 2689
Available specific dataset
n = 1322
Incomplete dataset
n = 1367
Willing to attend London-based clinic
n = 779
Unknown willingness
n = 302
Not willing to attend London-based clinic
n = 241
n=62
Invited for study participation
n = 841
Attended research centre
n = 502
Did not attend research centre
n = 339
Refusals n=204
Temporary refusals n=28
Not eligible n=69
Non-response n=12
Deaths notified n=3
Cancelled visits n=23
Completed scan
n = 471
No scan
n = 31
Available for cognitive analysis
n = 499
Dementia-free
n = 468
Dementia
n = 3
T1 QC fails n=3
FLAIR QC fails n=2
PET machine crashed n=5
Early termination of scan n=2
PET data corrupted n=1
Available for amyloid analysis
n = 457
Passed MRI QC
n = 463
Available amyloid-PET data
n = 457
Available for brain volume analysis
n = 445
Segmentation fail n=1
Erroneous segmentation of stroke/vascular abnormality/demyelination n=9
Passed BaMoS QC
n = 453
Available for WMHV analysis
n = 453
Includes participants with neurological/major psychiatric diagnoses: Clinical or cortical stroke n=13; Traumatic brain injury or previous neurosurgery n=2; Parkinson's disease or other neurodegenerative disease n=4; Epilepsy n=6; Major psychiatric disease n=4; previous encephalopathy n=1; MCI n=6

Supplemental results

Supplementary table 1 Comparison of clinical characteristics between individuals with missing imaging and BP data and those who completed scanning and had complete BP data. By design, no individuals were missing BP information at all time-points. 58 individuals were missing a single measurement, 11 individuals were missing a BP measurement at two time points and 2 individuals were missing a measurement at three time points. 2 individuals were missing *APOE*-ε4 data. Specific *n* are provided for a given variable. BMI, body mass index; BP, blood pressure; DBP diastolic blood pressure; IQR, interquartile range; MMSE, mini-mental state examination; n, number; NA, not applicable; PACC, Preclinical Alzheimer Cognitive Composite; SBP, systolic blood pressure; SD, standard deviation

Variable		Complete BP information and completed scanning (n=398)	Missing BP at any age but completed scanning (n=70)	Did not complete scanning protocol (n=31)
**Male:Female**		204:194	36:34	15:16
**Age at Insight 46 assessment, mean (SD)**		70·7 (0·7)	70·9 (0·7)	70·8 (0·6)
**Amyloid positive, n (%)**		72 (18·6)<br>n=388	11 (15·9)<br>n=69	NA
**Whole brain volume in ml, mean (SD)**		1101·2 (98·8)<br>n=378	1092·7 (96·1)<br>n=67	NA
**Mean hippocampal volume in ml, mean (SD)**		3·1 (0·3)<br>n=378	3·1 (0·3)<br>n=67	NA
**White matter hyperintensity volume in ml, median (IQR)**		3·2 (1·6, 6·9)<br>n=386	2·4 (1·1, 5·8)<br>n=67	NA
**Total intracranial volume in ml, mean (SD)**		1435·3 (132·5)<br>n=386	1426·5 (131·6)	NA
**MMSE /30, mean (SD)**		29·3 (0·9)	29·2 (1·0)	29·4 (1·0)
**PACC z score, mean (SD)**		0·03 (0·68)	-0·06 (0·73)	0·03 (0·73)
**Standardised childhood cognition, mean (SD)**		0·46 (0·63)	0·37 (0·67)	0·39 (0·65)
**SBP at 36 in mmHg, mean (SD)**		120·3 (13·7)	120·0 (14·4)<br>n=29	119·2 (13·6)<br>n=30
**SBP at 43 in mmHg, mean (SD)**		123·6 (13·7)	124·1 (14·5)<br>n=46	122·6 (12·6)
**SBP at 53 in mmHg, mean (SD)**		134·2 (18·9)	130·3 (20·6)<br>n=57	130·4 (16·2)<br>n=30
**SBP at 60-64 in mmHg, mean (SD)**		135·2 (17·0)	132·7 (17·0)<br>n=69	136·0 (14·5)
**SBP at 69 in mmHg, mean (SD)**		132·1 (15·3)	132·7 (19·8)	133·6 (17·6)<br>n=30
**DBP at 36 in mmHg, mean (SD)**		78·6 (9·5)	78·3 (11·0)	76·1 (7·9)
**DBP at 43 in mmHg, mean (SD)**		80·4 (9·2)	81·6 (9·6)	78·7 (9·9)
**DBP at 53 in mmHg, mean (SD)**		83·4 (11·8)	81·7 (12·1)	81·7 (10·7)
**DBP at 60-64 in mmHg, mean (SD)**		77·2 (9·5)	75·2 (9·6)	78·4 (7·6)
**DBP at 69 in mmHg, mean (SD)**		73·3 (10·0)	73·8 (11·0)	74·2 (10·2)
**Antihypertensive medication at age 36, n (%)**		7 (1·8)	0 (0·0)<br>n=32	0 (0·0)<br>n=30
**Antihypertensive medication at age 43, n (%)**		7 (1·8)	1 (1·9)<br>n=53	1 (3·2)
**Antihypertensive medication at age 53, n (%)**		51 (12·8)	3 (4·8)<br>n=62	3 (10·0)<br>n=30
**Antihypertensive medication at age 60-64, n (%)**		113 (28·4)	19 (27·1)	9 (29·0)
**Antihypertensive medication at age 69, n (%)**		159 (40·4)	23 (37·1)<br>n=62	12 (40·0)<br>n=30
**Smoking Status at age 68**	**Current smoker**	13 (3·3)	3 (4·3)	2 (6·5)
	**Ex-smoker**	60·6 (63·8)	48 (68·6)	22 (71·0)
	**Never smoker**	144 (36·2)	19 (27·1)	7 (22·6)
**Hypercholesterolaemia at age 70, n (%)**		314 (78·9)	57 (81·4)	27 (87·1)
**Diabetes mellitus at age 70, n (%)**		40 (10·1)	10 (15·2)	5 (16·7)<br>N=30
**BMI at age 70 in kg/m^2, mean (SD)**		27·6 (4·4)	27·7 (4·5)	30·4 (5·7)
**Adult socioeconomic position, n (%)**	**Non-manual (Class I-IIIN)**	349 (87·7)	49 (70·0)	26 (83·9)
	**Manual (Class IIIM-V)**	49 (12·3)	21 (30·0)	5 (16·1)
***APOE*-ε4 carrier (1 or 2 alleles), n (%)**		118 (29·8)<br>n=396	21 (30·0)	7 (22·6)
